# Selective translational usage of TSS and core promoters revealed by translatome sequencing

**DOI:** 10.1186/s12864-019-5650-0

**Published:** 2019-04-11

**Authors:** Hua Li, Ling Bai, Hongmei Li, Xinhui Li, Yani Kang, Ningbo Zhang, Jielin Sun, Zhifeng Shao

**Affiliations:** 1 State Key laboratory for Oncogenes and Bio-ID Center, School of Biomedical Engineering, Shanghai Jiao Tong University, 800 Dongchuan Road, Shanghai, 200240 China; 2 Instrumental Analysis Center, Shanghai Jiao Tong University, 800 Dongchuan Road, Shanghai, 200240 China; 3 Key Laboratory of Systems Biomedicine (Ministry of Education), Shanghai Center for Systems Biomedicine, Shanghai Jiao Tong University, 800 Dongchuan Road, Shanghai, 200240 China

**Keywords:** CAGE, Translatome sequencing, TSS profiling, Core promoter, Polysome selection

## Abstract

**Background:**

In mammals, fine-tuned regulation of gene expression leads to transcription initiation from diverse transcription start sites (TSSs) and multiple core promoters. Although polysome association is a critical step in translation, whether polysome selectively uses TSSs and core promoters and how this could impact translation remains elusive.

**Results:**

In this study, we used CAGE followed by deep sequencing to globally profile the transcript 5′ isoforms in the translatome and transcriptome of human HEK293 cells at single-nucleotide resolution. By comparing the two profiles, we identified the 5′ isoforms preferentially used in translatome and revealed a widespread selective usage of TSSs (32.0%) and core promoters (48.7%) by polysome. We discovered the transcription initiation patterns and the sequence characteristics that were highly correlated with polysome selection. We further identified 5804 genes significantly enriched or depleted in translatome and showed that polysome selection was an important contributing factor to the abundance of related gene products. Moreover, after comparison with public transcriptome CAGE data from 180 human tissues and primary cells, we raised a question on whether it is a widely adopted mechanism to regulate translation efficiency by changing the transcription initiation sites on the transcription level in cells of different conditions.

**Conclusions:**

Using HEK293 cells as a model, we delineated an indirect selection toward TSSs and core promoters by the translation machinery. Our findings lend additional evidence for a much closer coordination between transcription and translation, warranting future translatome studies in more cell types and conditions to develop a more intricate regulatory model for gene expression.

**Electronic supplementary material:**

The online version of this article (10.1186/s12864-019-5650-0) contains supplementary material, which is available to authorized users.

## Background

The flow of genetic information is tightly controlled at multiple levels to maintain proper phenotypes and achieve cellular fitness. The regulation of transcription, the first step in gene expression, has been extensively studied and its complexity has been elaborated mostly owing to highly efficient next-generation sequencing techniques [1–3]. Besides transcriptional regulation, it is now more evident that translational regulation on mRNA also has substantial control over gene expression by modulating mRNA translation, stability and localization [4, 5] Translational regulatory factors constitute a highly complex network to control protein product and output, thus playing critical roles in cellular metabolisms and tumorigenesis [6–9].

The noncoding part of mRNA, including the 5′ UTR (with the 5′ cap), the 3′ UTR and the poly(A) tail, is responsible for most translational regulation on gene expression. The 5′ UTR is of special importance to translational initiation where protein synthesis is principally regulated: during the initiation step in eukaryotes, eukaryotic initiation factors recruit the small ribosomal subunit (40S) to form a pre-initiation complex that scans the 5′ UTR to locate the start codon, after which the initiation factors are released and the large ribosomal subunit (60S) is recruited to form the elongation-competent 80S ribosome [10]. Several features in the 5′ UTR, such as the 5′ cap, secondary structure and length, are known to affect translation [11, 12]. Other important regulatory features, such as upstream AUGs (uAUGs), have also been studied in recent genome-wide studies [4, 13, 14].

Cap Analysis of Gene Expression (CAGE) is a powerful method widely used to profile the 5′ ends in organisms like human, fly and yeast [1–3, 15–18]. Accumulated CAGE data have clearly shown that a single gene can have highly heterogeneous 5′ ends (i.e., 5′ isoforms) in total RNA (i.e., transcriptome). This heterogeneity is one manifestation of the complex transcriptional regulation in eukaryotes: the transcription machinery employs diverse transcription start sites (TSSs) and multiple core promoters to precisely and dynamically regulate gene transcription [19–21]. Besides, selective usage of TSSs and core promoters in transcription could have great impact on translation, thus altering the abundance of protein products or even changing the related biological functions [11, 19–21]. By contrast, systematic investigation on 5′ ends of polysome-associated RNAs (i.e., translatome), which is more closely related to translation process and protein products, has only been performed in a very limited scale [4, 11, 22]. Given the importance of the 5′ UTR, the lack of information on the difference between translatome and transcriptome will limit our ability to decipher the sophisticated regulatory mechanisms in translation.

In this study, we employed CAGE followed by deep sequencing to globally profile the transcript 5′ isoforms in the translatome of human HEK293 cells at single-nucleotide resolution. This allowed us to precisely annotate the 5′ ends, portray the 5′ end distributions, define the 5′ UTR and identify core promoters used by polysome. By comparing them with the counterparts from HEK293’s transcriptome, we revealed selective usage of the TSS-derived 5′ ends by polysome, thus delineating an indirect selection toward TSSs and core promoters by the translation machinery. In addition, quantitative measurement of transcript abundance with CAGE allowed us to investigate the transcript enrichment after polysome selection, enabling the identification of highly enriched or depleted gene products in translatome. All these differences between transcriptome and translatome highlight the important roles of polysome in regulating gene expression, the interplay between transcription and translation and the necessity of developing a more intricate model to explain the underlining mechanisms.

## Results and discussion

### The landscape of 5′ transcript ends in translatome and transcriptome

**Fig. 1:**
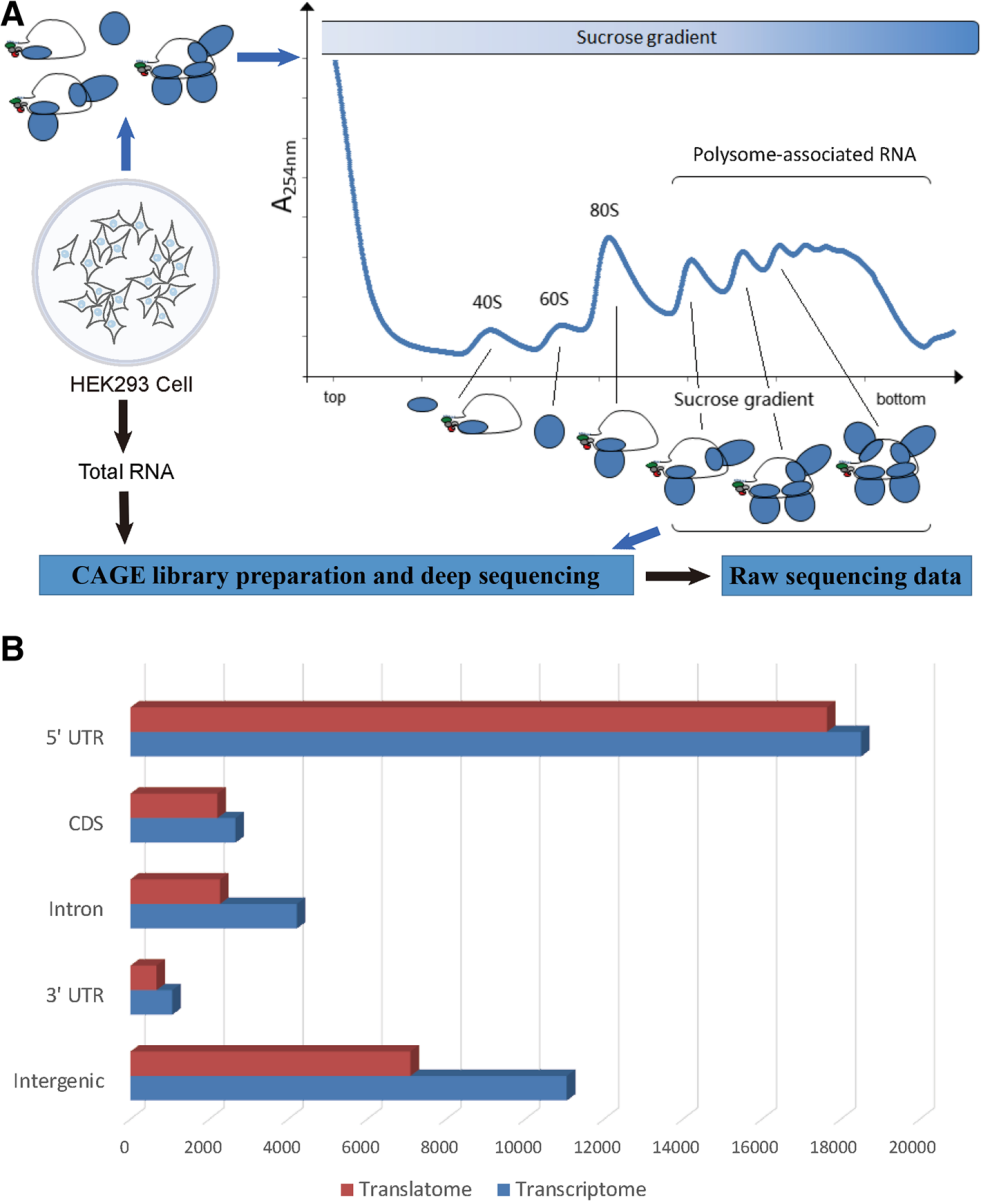
A flowchart of our work and the distribution of TCs. **a** A flowchart that briefly describes the steps to obtain the raw sequencing data. **b** Distribution of TCs across different genomic features in HEK293 cell line. Four genomic features (5′ UTR, 3′ UTR, CDS and intron) of all protein-coding genes were based on human GRCh37 annotations from Ensembl. The rest of the genome were considered “intergenic” (see Methods)

A flowchart of our work before data analysis is shown in Fig. 1a. CAGE tags from deep sequencing were processed with fqtrim to remove low-quality ones, generating approximately 18 million and 14 million tags respectively for the translatome and transcriptome of HEK293 [23]. These tags were then mapped to the human genome (assembly GRCh37) using bowtie with two mismatches allowed [24]. Tags mapped to rRNA were less than 17.9% for translatome and 7.5% for transcriptome, indicating high quality of the two CAGE libraries [3, 16]. In total, 6,973,108 tags (37.9%) for translatome and 6,791,846 tags (49.3%) for transcriptome were uniquely mapped and used for downstream analysis. The vast majority of CAGE tags were located within 100 nt flanking the 5′ ends of known transcripts in both translatome (79.4%) and transcriptome (72.0%) (Additional file 1: Figure S1), which was consistent with previous studies [16, 22, 25]. All tags were mapped to 804,594 and 1,315,195 unique genomic positions, with top 100,000 positions (< 0.01% of the human genome) representing 83.0 and 70.9% of all tags in translatome and transcriptome, respectively. These results showed a significant aggregation of CAGE tags and an excellent agreement between our data and existing annotations. They also showed that, the number of unique 5′ ends in translatome is much less than in transcriptome, which was a highly expected result.

We merged overlapped CAGE tags (at least 4 tags) into tag clusters (TC) and each TC represents a putative core promoter (see Methods) [3, 16]. In total, we identified 29,908 and 37,530 TCs, consisting of 97.0 and 90.9% CAGE tags in translatome and transcriptome, respectively. Using the 5′ end of the most redundant tag in a TC to represent the position of the TC, we calculated the distribution of TCs across four annotated genomic features of all protein-coding genes (Fig. 1b; unless otherwise specified, we used “genes” hereafter to refer to protein-coding genes only). In translatome, we observed a much higher proportion (59.0%) of TCs located within 5′ UTRs than in transcriptome (49.3%; *p*-value < 0.001 by proportion test), suggesting that transcripts with canonical ORFs were more likely to be translated. We identified a considerable fraction of TCs from intron and the coding sequence (CDS) in transcriptome (similar to the findings in human [16, 22, 25]; Fig. 1b), most of which were also present in translatome. Although it is unclear what proportion of these TCs would be further translated, recent studies have already highlighted the biological significance of the resulting truncated peptides [19, 20, 26, 27].

### Selective usage of TSS by Polysome

**Fig. 2:**
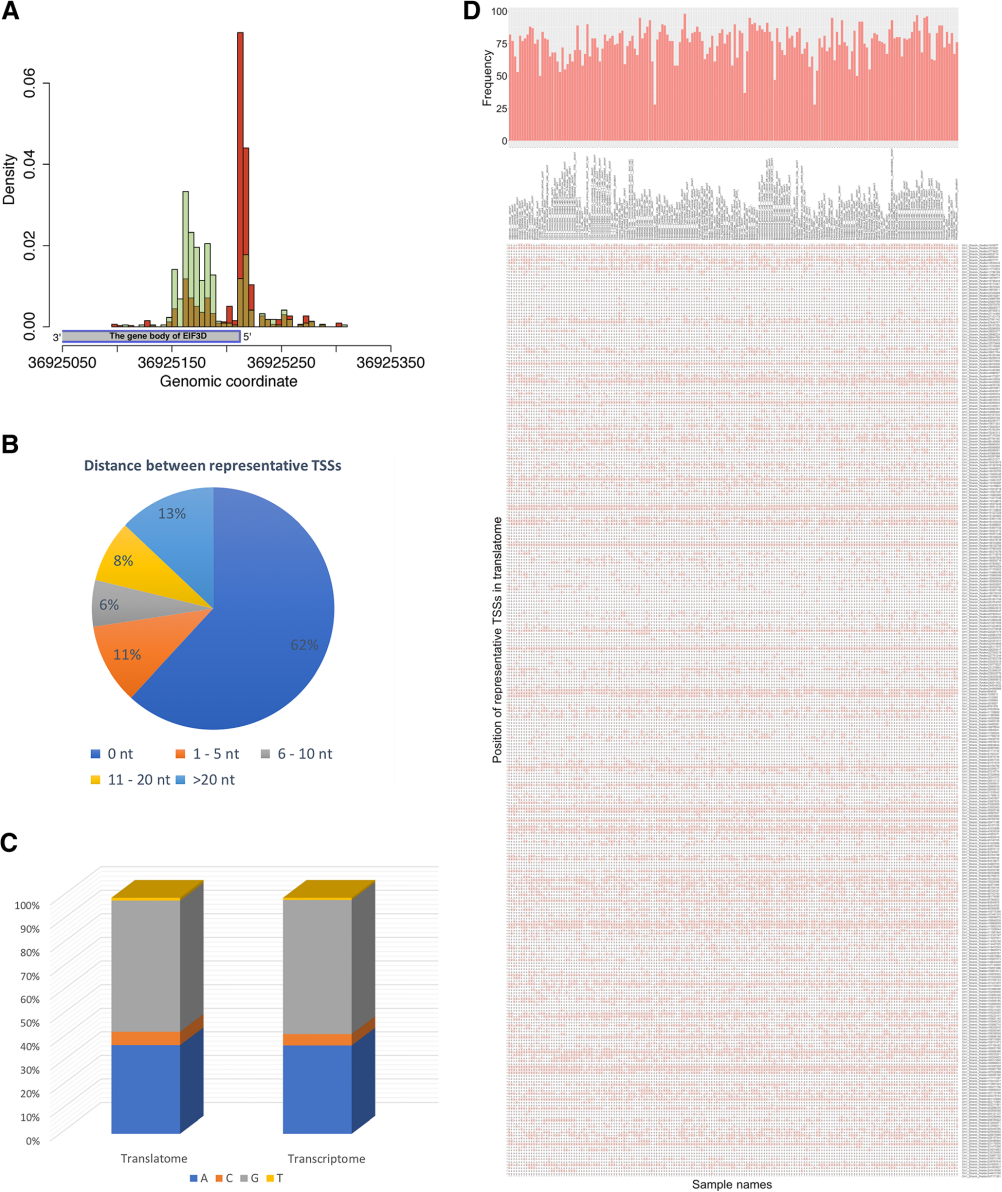
Comparison of TSSs between translatome and transcriptome. **a** A typical example of TSS distribution disparity. The two TCs are located in at the core promoter region of the gene “EIF3D”. The red color stands for the 5′ end distribution in translatome and the green stands for the distribution in transcriptome (the yellow is the common part of the two distributions). **b** The distance between representative TSSs of translatome and transcriptome. The distance is calculated with the genomic coordinates of representative TSSs on the human genome. **c** Comparison of the nucleotide frequency at representative TSSs in translatome and transcriptome. **d** Usage landscape of HEK293-derived representative TSSs in 180 human samples. All 3248 representative TSSs are from HEK293 translatome and only those from chromosome 1 are displayed here for better visualization. In the heatmap, “1” (marked with red) means the representative TSS on the right side is also used as representative TSS in the sample on the top. The histogram on top of sample names shows the number of representative TSSs used by each sample. The name of each representative TSS contains the information of chromosome, strand and genomic position. For the full list of all 3248 representative TSSs, please refer to Additional file 5: Table S3

In human, transcription usually initiates from multiple positions (i.e., TSSs) within core promoters, resulting in diverse distribution patterns of 5′ transcript ends in transcriptome [3, 16, 25, 28]. As expected, using a method proposed in previous studies [3, 16], we were able to classify the TCs of transcriptome into 4 shape classes based on the 5′ end distributions (Additional file 2: Figure S2). The 4 classes are (*i*) single dominant peak (SP), (*ii*) broad with a single dominant peak (DP), (*iii*) broad with bi- or multi- peaks (MP), and (*iv*) generally broad peaks (BP), the proportions of which are comparable to previous studies [16, 25]. We hereafter only analyzed TCs located in the annotated 5′ UTRs (including the upstream 100 nt) since these TCs and their internal 5′ ends corresponded to core promoters and TSSs, respectively [3, 16]. Very importantly, although we were also able to classify the TCs of translatome into these 4 classes, the 5′ end (i.e., TSS) distributions for a large proportion of TCs changed from their counterparts in transcriptome. This distribution disparity was assessed using Kolmogorov-Smirnov test (KS test) for TCs with at least 100 tags in both translatome and transcriptome. To our surprise, as many as 1781 TCs (32.0%) underwent 5′ end distribution change (*p*-value < 0.001 by KS test; Fig. 2a), of which 775 (43.5%) had different shape classes between translatome and transcriptome (Additional file 3: Table S1). These results demonstrate that preferential usage of TSSs by polysome is a widespread phenomenon in HEK293 cell line.

The position of the highest density in the 5′ end distribution, which corresponds to the most frequently used TSS (defined as representative TSS) within a core promoter, was used in previous work to define the 5′ UTR [3]. In this study, representative TSSs and the 5′ UTR were defined in the same way, and length difference of the 5′ UTR was calculated between translatome and transcriptome (Additional file 4: Table S2). Among the 8513 TCs with at least 10 tags mapped to the representative TSSs, 38.2% (3248) had different representative TSSs between translatome and transcriptome (Fig. 2b); by contrast, only 20.3% of the 8513 TCs (much lower than 38.2%; *p*-value < 0.001 by proportion test) used different nucleotides at the representative TSSs, showing conservations that were consistent with previous findings that the identity of the first nucleotide had a strong influence on the translation of the corresponding transcript [20]. In addition, in transcriptome, the nucleotide frequencies at the representative TSSs were similar to those in previous studies [16, 25]; in translatome, the frequencies were largely the same, except that the frequency of “C” increased appreciably (1.2-fold change, p-value < 0.05 by proportion test; Fig. 2c).

We downloaded deep sequencing-derived CAGE data from the FANTOM5 project (phase 1.3 and 2.0), which included the TCs and representative TSSs in transcriptome for 180 human tissues and primary cells [29]. We compared the representative TSSs between the HEK293 translatome and the 180-sample CAGE data. We found that, almost 90% of the aforementioned 3248 representative TSSs could be found in at least 1 of the 180 transcriptome CAGE data as representative TSSs (Fig. 2d; Additional file 5: Table S3). Since the representative TSSs in translatome shows the most preferred 5′ transcript ends by polysome, exactly matched representative TSSs between transcriptome and translatome should contribute to better efficiency for polysome association, thus probably enhancing translational efficiency. Therefore, based on these observations, we raised a question worth further investigations: could it be a widely adopted mechanism to regulate translation by employing different representative TSSs at the transcription level in different cell types?

### Selective usage of core promoters by Polysome

**Fig. 3:**
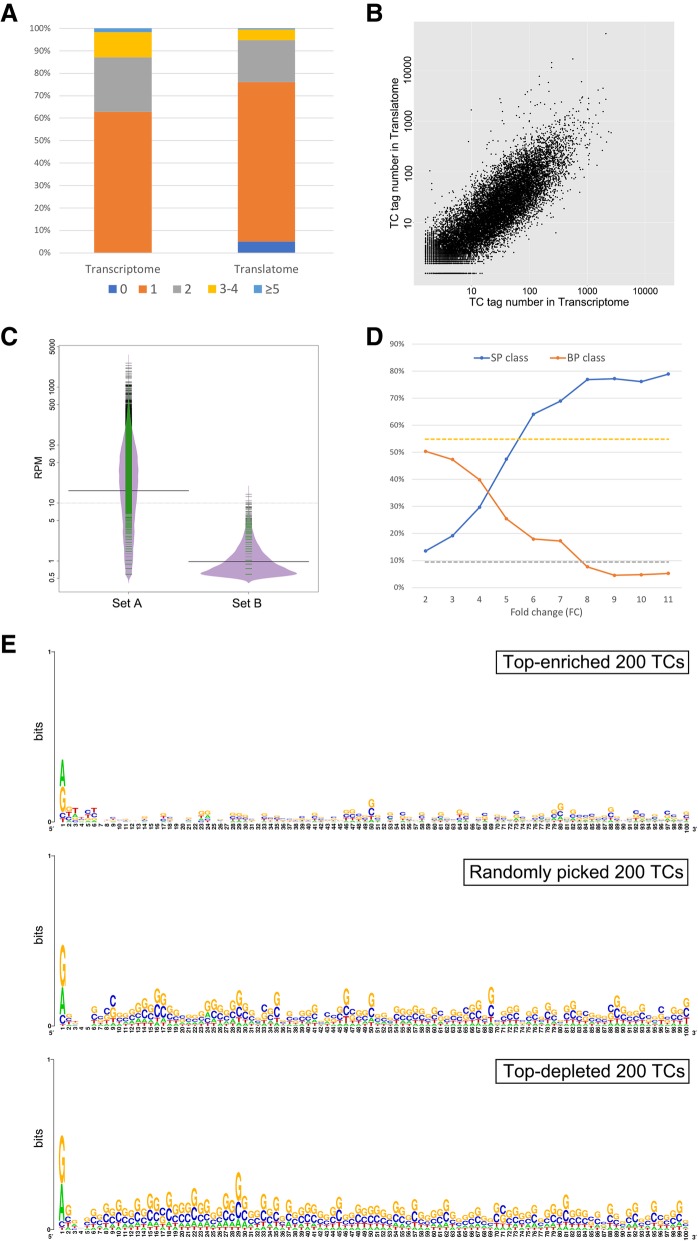
Comparison of core promoter usage in translatome and transcriptome. **a** The percentage of genes using specific number of core promoters. **b** Scatterplot of the TC tag numbers in translatome and transcriptome. As the two axes are in logarithmic scale, the number of tags in all TCs has been increased by 1 to avoid 0. **c** Beanplot of the TC abundance measured by RPM. Among the TCs in transcriptome, those present in translatome are grouped into “Set A” while those not present are grouped into “Set B”. In this plot, the short green lines mark the observations of RPM, while the purple area shows the frequency of the observed RPM. The two long solid black lines stand for the average of each set and the long dotted black line stands for the overall average of two sets. **d** Correlation between TC fold change and TC shape class. The percentage (y-axis) of SP and BP classes is calculated with translatome-enriched TCs with minimal fold change on x-axis. The dashed grep line and yellow line stand for the percentage of SP and BP classes for translatome-depleted TCs with fold change < 0.5. **e** The consensus sequence of the immediate downstream 100 nt of the representative TSSs of the top-enriched 200 TCs, top-depleted 200 TCs and randomly picked 200 TCs. The three groups of 100-nt sequences were all analyzed with WebLogo. The x-axis shows the relative positions with respect to the representative TSS (at position 1)

In eukaryotes from yeast to human, a single gene could use multiple core promoters in transcription as a result of complex gene expression regulation [3, 16, 25]. Here in HEK293 cell line, 37.2% of the expressed genes used at least 2 core promoters to initiate their transcription (Fig. 3a); by contrast, the percentage of genes still using ≥2 core promoters went down significantly in translatome (25.3%, *p*-value < 0.001 by proportion test). Although the majority of core promoter-derived TCs were also associated with polysome (Fig. 3b), their abundance (measure by Reads Per Million – RPM) could be changed significantly on polysome (see the next paragraph). An unneglectable fraction (17.5%) of core promoter-derived TCs were not present in translatome, and their average RPM were much lower than that of the others in transcriptome (*p*-value < 0.001 by Wilcoxon test; Fig. 3c). These results suggest that preferential usage of core promoters is a common phenomenon for polysome in HEK293 cell line.

We found a surprisingly high proportion (48.6%) of TCs with significantly changed RPM (|log_2_FC| ≥1 and *p*-value < 0.05 by the R package of DEGseq, where FC (i.e., fold change) is defined as RPM_translatome_/RPM_transcriptome_ for each TC) between translatome and transcriptome [30]. Among these TCs (Additional file 6: Table S4), only 2488 (25.8%) showed enrichment in translatome, corroborating the preferential usage of certain core promoters in translation (see Additional file 7). Moreover, for translatome-enrich TCs, we discovered a significant correlation between transcription initiation pattern and TC enrichment level (Fig. 3d): when the TC fold change went higher, the percentage of TCs in SP class generally went much higher (Pearson correlation *R* = 0.94, *p*-value < 0.001) while the percentage of BP class generally became much lower (*R* = -0.95, *p*-value < 0.001). Since the shape class of the same TC may change between different types of cells [25, 28, 29], it could be a potential strategy to regulate translation by controlling the transcription initiation pattern.

We compared the immediate downstream sequences (100 nt) of representative TSSs between top-enriched 200 TCs, top-depleted 200 TCs and randomly picked 200 TCs with FC between 0.95 and 1.05. We first used WebLogo to examine the sequence features and found that the top-enriched TCs had the least GC content while the top-depleted ones had the most (Fig. 3e) [31]. By counting the total number of AUG (the start codon) in the 100 nt sequence for each group, we found that the top-enriched TCs had significantly more AUG (151) than the top-depleted (78) and the randomly picked (97) groups (*p*-value < 0.001 by proportion test), showing that the polysome preferentially binds to transcripts with shorter 5′ UTR. Sequence motif analysis with MEME identified a significant motif AA(G/A)(A/C)A(G/A)GG in the downstream 100 nt sequences for the top-enriched 200 TCs (p-value< 0.001), which was not present in the other two groups [32]. Further analysis using Tomtom showed that this motif was not similar to any protein-binding motif (*p*-value > 0.05), indicating it could be a novel motif [33]. Importantly, compared with the other two groups, there was a significant enrichment (> 6 fold) of TATA box in the upstream 100 nt of the top-enriched 200 TCs, suggesting this cis-regulatory element played important roles beyond transcription regulation.

### Selective usage of genes by Polysome

Suppose a gene *A* could generate transcripts from *n* different core promoters (*n* is determined by GRCh37 annotations from Ensembl), we used {*p*_1_, *p*_2_,  … , *p*_*n*_} and {*t*_1_, *t*_2_,  … , *t*_*n*_} to denote the abundance of core promoter-derived TCs in translatome and transcriptome, respectively. We first defined gene *A*’s abundance in translatome (*E*_*p*_) and transcriptome (*E*_*t*_) as follows:

$$ {E}_p={\Sigma}_{i\kern0.5em =\kern0.5em 1}^n\kern0.5em {p}_i;\kern0.5em {E}_t\kern0.5em =\kern0.5em {\Sigma}_{i\kern0.5em =\kern0.5em 1}^n\kern0.5em {t}_i $$

We then defined a fold-change score (*S*_*fc*_) as follows to measure polysome preference toward genes:

$$ {S}_{fc}\kern0.5em =\kern0.5em \frac{E_p}{E_t}\kern0.5em =\kern0.5em \frac{\Sigma_{i\kern0.5em =\kern0.5em 1}^n\kern0.5em {p}_i}{\Sigma_{i\kern0.5em =\kern0.5em 1}^n\kern0.5em {t}_i} $$

We calculated *S*_*fc*_ for each gene and the corresponding *p*-value with the DEGseq package (Additional file 8: Table S5). In total, we identified 5804 (49.5%) genes with significantly changed abundance (i.e., |log_2_ *S*_*fc*_ | ≥1 and *p*-value < 0.05). We then looked into the top 50 translatome-enriched genes (all *S*_*fc*_ > 2) ranked by the *p*-values (Table 1) and found that the translatome-enriched ones were highly enriched in the gene families of histones and ribosomal proteins (both p-values < 0.001 by fisher’s exact test). By contrast, the top 50 translatome-depleted genes (all *S*_*fc*_ < 0.5) enriched in the RNA binding motif containing genes (p-value < 0.001), included no histone or ribosomal genes. Considering that histones and ribosomal proteins are highly abundant in cells and polysome association is a prerequisite for translation [10, 34], we infer that polysome selection is an important contributing factor to the abundance. To support this point, we picked three groups of genes (translatome-enriched, translatome-depleted and unchanged) with similar average RPM in transcriptome (see Methods). We found that the protein abundance of translatome-enriched genes was significantly higher than the other two groups (p-value < 0.001 by Wilcoxon test), while enrichment-unchanged group had much higher abundance than that of translatome-depleted genes (p-value < 0.001), thus substantiating our inference (see Methods).

**Table 1:**
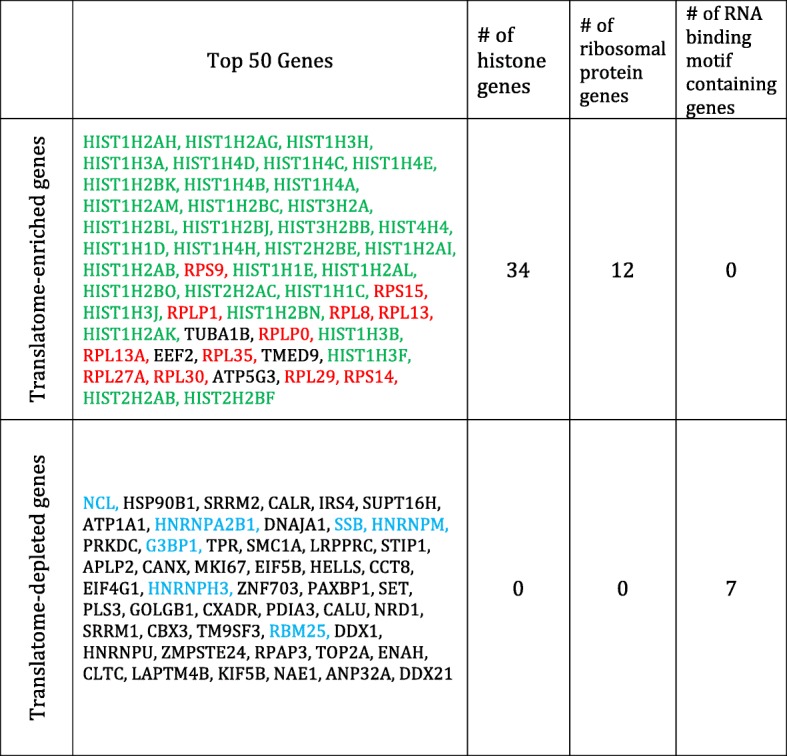
The top 50 genes that are most enriched or depleted in translatome. Genes are ranked by *p*-values (all p-values < 0.001). The histone genes are marked in green, the ribosomal protein genes are in red and the RNA binding motif containing genes in blue. “#” stands for “number”

As differential usage of core promoters from the same gene could have profound impact on the protein products and the related biological functions [19, 21], we formulated another score (*S*_*du*_) as follows to measure the degree of differential usage:

$$ {S}_{du}\kern0.5em =\kern0.5em \frac{1}{2}\kern1em \underset{i\kern0.5em =\kern0.5em 1}{\overset{n}{\Sigma}}\mid \frac{p_i}{E_p}\kern0.5em -\kern0.5em \frac{t_i}{E_t}\mid $$

This equation applies only when gene *A*’s abundance is > 0 in both translatome and transcriptome (i.e., *E*_*p*_ > 0 and *E*_*t*_ > 0). Based on this equation, we could easily conclude that: (1) *S*_*du*_ ∈[0, 1]; (2) *S*_*du*_ = 0 when gene *A* only uses 1 core promoter (i.e., *n* = 1), or uses ≥2 core promoters (i.e., *n* ≥ 2) but the core promoter-derived TCs account for the same proportions between translatome and transcriptome (i.e., $\frac{p_i}{E_p}=\frac{t_i}{E_t}$ given that 2 ≤ *i* ≤ *n*); (3) *S*_*du*_ = 1 when gene A only uses TCs (in translatome) that are not detected in transcriptome by the Illumina sequencing. For simplicity, we only calculated *S*_*du*_ for genes using at least 2 core promoters (i.e., *n* ≥ 2) in HEK293 cell line. Importantly, we found that, the correlation between *S*_*du*_ and *S*_*fc*_ is very low (Pearson correlation *R* = -0.04), demonstrating that *S*_*du*_ give additional information independent of *S*_*fc*_. We identified 62 genes with *S*_*du*_ > 0.5 and further analysis of them showed that they were enriched in the myocardin gene family (*p*-value < 0.001 by Fisher’s exact test). By contrast, the top 62 genes with smallest *S*_*du*_ were only enriched in the gene families of histones and ribosomal proteins (p-value < 0.001), suggesting that differential usage of core promoters was very rare for histone and ribosomal genes in HEK293 cell line. Here we listed > 4700 genes (including the aforementioned 62 × 2 genes) with their *S*_*du*_ scores in Additional file 9: Table S6 to spur interest of biologists for the underlining mechanism leading to this differential usage.

## Conclusions

In this work, we use CAGE followed by deep sequencing to systematically compare the transcript 5′ ends between the translatome and transcriptome of human HEK293 cells. The revealed preferential usage of many 5′ ends by polysome shows that, after transcriptional selection of TSS and core promoters, the translation machinery again makes such selection. This comparison leads to the identification of highly selected TSSs, core promoters and gene products in translatome. It also gives rise to the transcription initiation patterns and the sequence characteristics highly correlated with polysome selection. These findings delineate an indirect selection toward TSSs and core promoters by the translation machinery, emphasizing closer than expected interplay between transcription and translation.

## Methods

### Growth conditions and RNA isolation

HEK293 cells were cultured in Dulbecco’s Minimal Essential Medium (GIBCO, Life Technologies, Carlsbad, CA, USA) supplemented with 10% FBS (GIBCO #10099–141), 100 units/ml penicillin, 100μg/ml streptomycin (GIBCO #15140–122) and 2 mM L-glutamine (Sigma) at 37 °C and 5% CO_2._

Polysome fraction is isolated by 10–50% sucrose gradient using the method from Bor et al. (2006) with minor modifications [35]. In brief, around 80% confluent cells were incubated with 50 μg/ml cycloheximide for 30 min at 37 °C. Cells were scrapped into a 1.5 ml Eppendoff tube with a cell lifter. And cells were lysed by 250ul 2XRSB/RNasin and 250 μl of polysome extraction buffer. The polysome fraction was collected with the BioComp piston gradient fractionator after ultra-speed centrifugation with SW41Ti rotor buckets at 36,000 rpm for 2 h.

The cells without cycloheximide treating were lysed with TRIzol reagent (Invitrogen, Cat. No. 15596–018) and total RNA was extracted following TRIzol protocol. The polysome-associated RNA was also extracted using TRIzol with the same method.

### CAGE library preparation

Two 27 bp-tagged deep sequencing libraries were prepared using the methods described in Valen et al. (2009) and Takahashi et al. (2012) [17, 36]. In brief, using SuperScript II (Invitrogen), first-strand cDNA was synthesized with 30 μg total RNA and 8 μg of anchored-N15 primer(5′- AAGGTCTATCAGCAGN15). The capped RNA was selected using the cap-trapper method described in Valen et al. (2009) [17]. The 2nd strand DNA was synthesized by ligating a N6 adaptor (CCACCGACAGGTTCAGAGTTCTACAGCTTCAGCAGNNNNNN Phos / N6-down: Phos CTGCTGAAGCTGTAGAACTCTGAACCTGTCGGTGG NH2) to the 3’end of the ssDNA with DNA ligation kit (Takara, Tokyo, Japan). The dsDNA was digested with EcoP15I (NEB, Ipswich, MA, USA) following the method from Takahashi et al. (2012) [36]. A 3′ adaptor (3′ adaptor-up, NNTCGTATGCCGTCTTCTGCTTG / 3′ adaptor-down: CAAGCAGAAGACGGCATACGA) was ligated to the recovered EcoP15I fragments. The PCR primer1 (5′- CAAGCAGAAGACGGCATACGA -3′) and PCR primer2 (5′-AATGATACGGCGACCACCGACAGGTTCAGAGTTCTACAGTCCGA -3′) were used to create deep sequencing libraries which contained the first 27 bp from the 5′ ends of capped RNA. The two libraries were prepared and sent out for sequencing together. Sequencing was performed using Solexa GAII following the manufacturer’s protocol. Sequencing data from different batches (to achieve enough sequencing depth) were merged together before downstream data analysis.

### Quality control and sequencing reads alignment

Raw sequencing data were first processed using fqtrim (version 0.94) to remove the 3′ sequencing adaptor, 5′ barcodes (CTTCAGCAG and GATCAGCAG for translatome and transcriptome RNA library respectively) and low-quality reads (with parameters -q 20 -m 1) [23, 27]. Reads with length ≥ 30 or ≤ 24 were also removed from downstream analysis since the expected length of CAGE tags was 27 nt based on the protocols above [35, 36]. Bowtie was then used to map the clean reads to the human genome (assembly GRCh37) with two mismatches allowed (using parameters -v 2 --best --strata -m 1). Only uniquely mapped reads were used for further analysis. The R package – CAGEr was used to correct “G” nucleotide addition bias at the 5′ ends of CAGE tags introduced in the library preparation [28].

### Tag clustering and TC distribution

Tag clusters were identified with the following two steps. First, tags that overlapped on the same strand were grouped into a tag set. Second, any tag set with at least four tags were defined as a tag cluster. Suppose tags randomly distributed on the human genome were background noise, the probability *n* tags were observed in a tag set of (*n*-1) × 27 nt length follows a Poisson distribution with $\lambda =\frac{\left(n-1\right)\times 27}{N}\times T$ (*N* is the human genome length and *T* is the number of uniquely mapped reads). Based on this λ, it is obvious that the probability of *n* ≥ 4 is less than 0.001, which corresponds to the *p*-value. Therefore, the above two steps guaranteed that the identified tag clusters had significant *p*-values and were not background noise.

Different genomic features could overlap and the positions of TCs could be situated within two or more features at the same time. When this happened, we used the method described in Ref. [3] to assign TCs with the following priority: 5′ UTR > 3′ UTR > CDS > intron (the 100 nt upstream of 5′ UTR were also included in 5′ UTR) [3]. TCs not mapped to any of the four features in protein-coding genes were considered “intergenic”.

### Classification criteria for TC shape class

We classified TCs (with ≥100 tags) into four shape classes with a method similar to those from previous studies [3, 16]. Briefly, we used the following criteria: (*i*) a TC was classified into SP class if the distance between the 25th and 75th percentile of its tag positions was < 4 nt, or the distance between the 15th and 85th percentile was < 6 nt; (*ii*) if a TC did not meet (*i*) but the ratio between its highest peak and second highest peak was > 2 and the highest peak accounted for > 20% of all tags in it, the TC was classified into DP class; (*iii*) If distance between any two consecutive peaks (both peaks accounted for > 15% of all tags) was > 5 nt and the TC was in neither SP nor DP class, it was classified into MP class; (*iv*) if a TC did not meet (*i*), (*ii*) or (*iii*), the TC was classified into BP class.

### Additional definitions, tools and data sources

In this study, the expressed transcripts were defined as those with at least one TC identified within the 5′ UTR or the 100 nt upstream region. The expressed genes were defined as those with at least one annotated transcript expressed in transcriptome. To make comparison between samples, RPM was used to normalize the read number of each cluster, which was defined as follows:

$$ \mathrm{RPM}=\frac{the\ number\ of\ reads\ in\ a\  read\ cluster}{the\ total\ number\ of\ mapped\ reads}\times 1000000 $$

Statistical analysis (including the hypothesis testing) was performed with the R language (http://www.r-project.org/). In the case of multiple hypothesis testing, we used BH method to correct *p*-values (unless otherwise specified) [37]. Sequence and motif analysis was performed based on R, WebLogo (http://weblogo.berkeley.edu) and MEME (http://meme-suite.org/tools/meme, with the option “search given stand only” for motif search only in the RNA transcripts) and Tomtom [http://meme-suite.org/tools/tomtom, with Vertebrates (In vivo and in silico) as the database of known motifs] [31–33]. Multiple R packages and tools were used in DNA sequence retrieval and figure preparation [38–43].

All CAGE data from the FANTOM5 project were downloaded from the FANTOM website (http://fantom.gsc.riken.jp/5/datafiles/latest/), including the TCs and the representative TSSs for 180 human tissues and primary cells (see Additional file 9: Table S6 for more details of the 180 samples) [29]. The category of gene families and their members were retrieved from HGNC website (https://www.genenames.org/) [44].

### Polysome selection and protein abundance

We picked three groups of genes with the following criteria: (1) picked top 100 genes from the translatome-enriched genes (ranked by p-values; all *S*_*fc*_ > 2); (2) picked top 100 genes from the unchanged genes (i.e., 0.9 < *S*_*fc*_ < 1.1; genes were ranked by their RPM in transcriptome); (3) picked the genes ranked from 101th to 200th from the translatome-depleted genes (ranked by p-values; all *S*_*fc*_ < 0.5). This way, we obtained three groups consisting of 100 genes each with similar average RPM in transcriptome (290.3, 298.8, 300.4 for translatome-enriched, unchanged, translatome-depleted genes, respectively). We retrieved protein abundance data (i.e., average protein copy number) in mouse fibroblasts (NIH3T3 cell line) from Ref. [34] Additional file 5: Table S3. We identified the homologous proteins between mouse and human based on HGNC nomenclature and used mouse proteins’ abundance to represent the homologs’ abundance in human [44].

### Accession number

Raw sequencing data used in this work are available in the ArrayExpress database (http://www.ebi.ac.uk/arrayexpress) under accession number E-MTAB-7382.

## Additional files

Additional file 1: **Figure S1.** Tag distribution around annotated TSSs of human transcripts. The black line stands for translatome and the red line stands for transcriptome. The TSS annotation was retrieved from human GRCh37 annotations downloaded from Ensembl. (PNG 188 kb)

Additional file 2: **Figure S2.** Typical examples for the 4 TC shape classes. SP class (A) are characterized by a sharp peak that stands for the majority of tags in a TC. BP class (D) do not have any peak much stronger than the others in a TC. The 5’ end distributions in DP (B) and MP (C) classes are somewhere between SP and BP classes (refer to Methods for more details). The TC information (chromosome, strand and genomic position) are placed on top of each example. (PNG 336 kb)

Additional file 3: **Table S1.** TCs with changed 5’ end distributions between translatome and transcriptome. “Chr” stands for “chromosome”. “TC start” and “TC end” gives the genomic range of TCs on the human genome. “Gene Symbol” shows the genes where TCs are located. “Source” shows where TCs come from. *P*-values are calculated with KS test and adjusted with BH method. Any two TCs in sequential rows from Translatome and transcriptome correspond to the same core promoter and thus have the same *p*-value. (XLSX 268 kb)

Additional file 4: **Table S2.** Length difference of the 5’ UTR between translatome and transcriptome. “Length Difference” shows the difference in length of the 5’ UTR. “Length Status” shows whether the length of the 5’UTR in translatome is the same as, or shorter than, or longer than that in transcriptome. The definitions of the other column names are the same as in Additional file 3: Table S1. (XLSX 1009 kb)

Additional file 5: **Table S3.** Usage frequency of HEK293-derived representative TSSs in 180 human samples. All 3248 representative TSSs are from HEK293 translatome. In the table, “1” means the representative TSS (row name) is also used as representative TSS in the sample (column name). The name of each representative TSS contains the information of chromosome, strand and genomic position. (XLSX 1773 kb)

Additional file 6: **Table S4.** TCs with significantly changed RPM between translatome and transcriptome. “z-score”, “p-value” and “q-value” are calculated with the R package of DEGseq. TCs are ranked by the q-values. (XLSX 2126 kb)

Additional file 7: Calculation of TC fold change (FC) with polysome-free RNA instead of total RNA. (DOCX 13 kb)

Additional file 8: **Table S5.** Comparison of genes’ abundance between translatome and transcriptome. “*E*_*p*_”, “*E*_*t*_” and “*S*_*fc*_” are all defined in the main text. “p-value” and “q-value” are calculated with the R package of DEGseq. Genes are ranked by the q-value. Genes with too few reads to calculated the *p*-values are removed from this table. (XLSX 925 kb)

Additional file 9: **Table S6.** Differential usage of core promoters from the same gene by polysome. Under each gene name, there are two (or more) rows corresponding to two (or more) core promoters of the gene. Each gene has one *S*_*du*_ score, which is defined in the main text. (XLSX 660 kb)

## Abbreviations

CAGE: Cap Analysis of Gene Expression
CDS: Coding sequence
FC: Fold change
KS test: Kolmogorov-Smirnov test
RPM: Reads per million
TC: Tag cluster
TSS: Transcription start site
UTR: Untranslated region
