# Supplementary figures and images for: Selective translational usage of TSS and core promoters revealed by translatome sequencing
Source: BMC Genomics. 2019 Apr 11;20:282. doi: 10.1186/s12864-019-5650-0 (PMC6463679; doi:10.1186/s12864-019-5650-0)

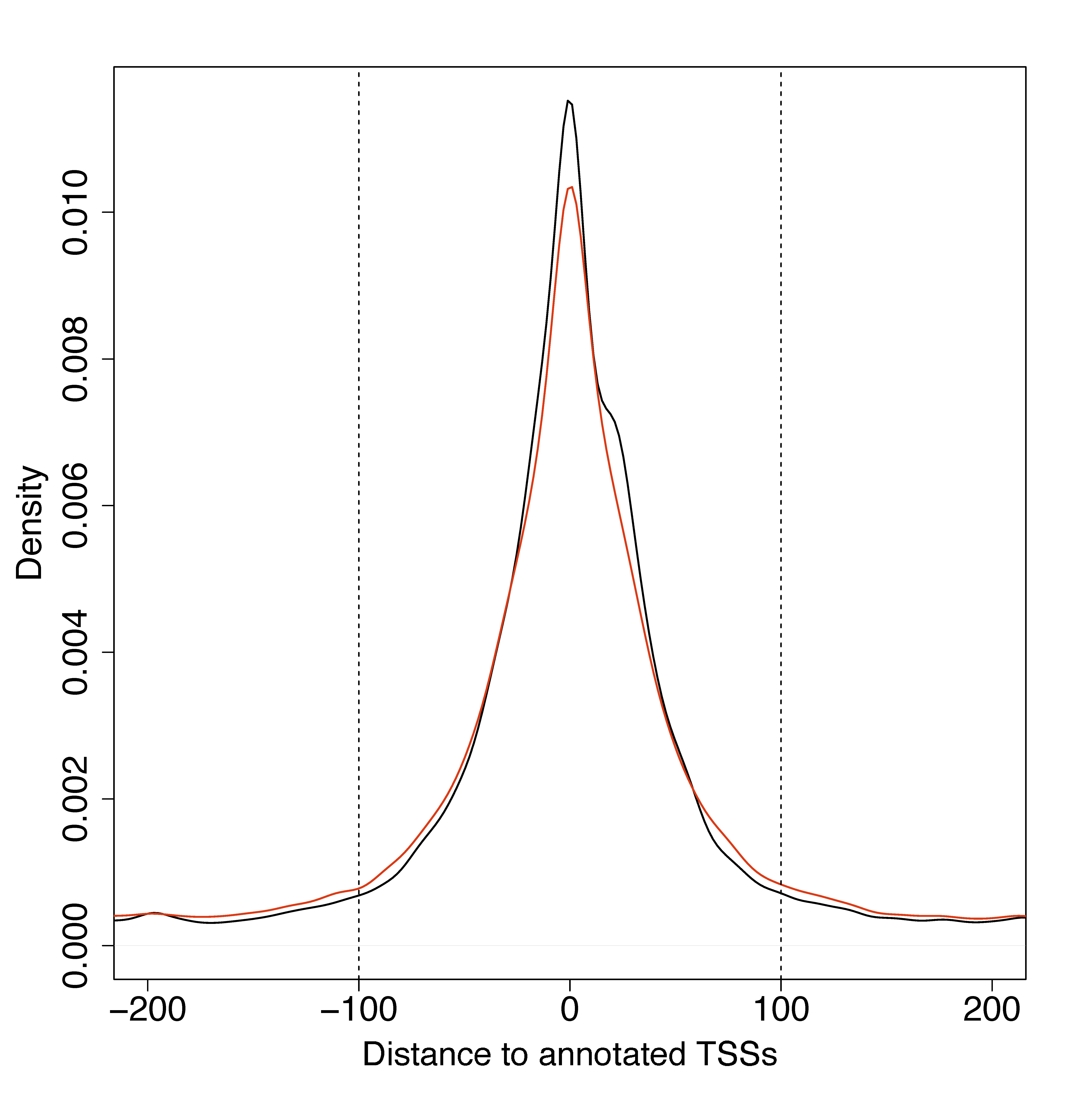

Supplement: Supplementary file 1 — Figure S1. Tag distribution around annotated TSSs of human transcripts. The black line stands for translatome and the red line stands for transcriptome. The TSS annotation was retrieved from human GRCh37 annotations downloaded from Ensembl. (PNG 188 kb) [file 12864_2019_5650_MOESM1_ESM.png]

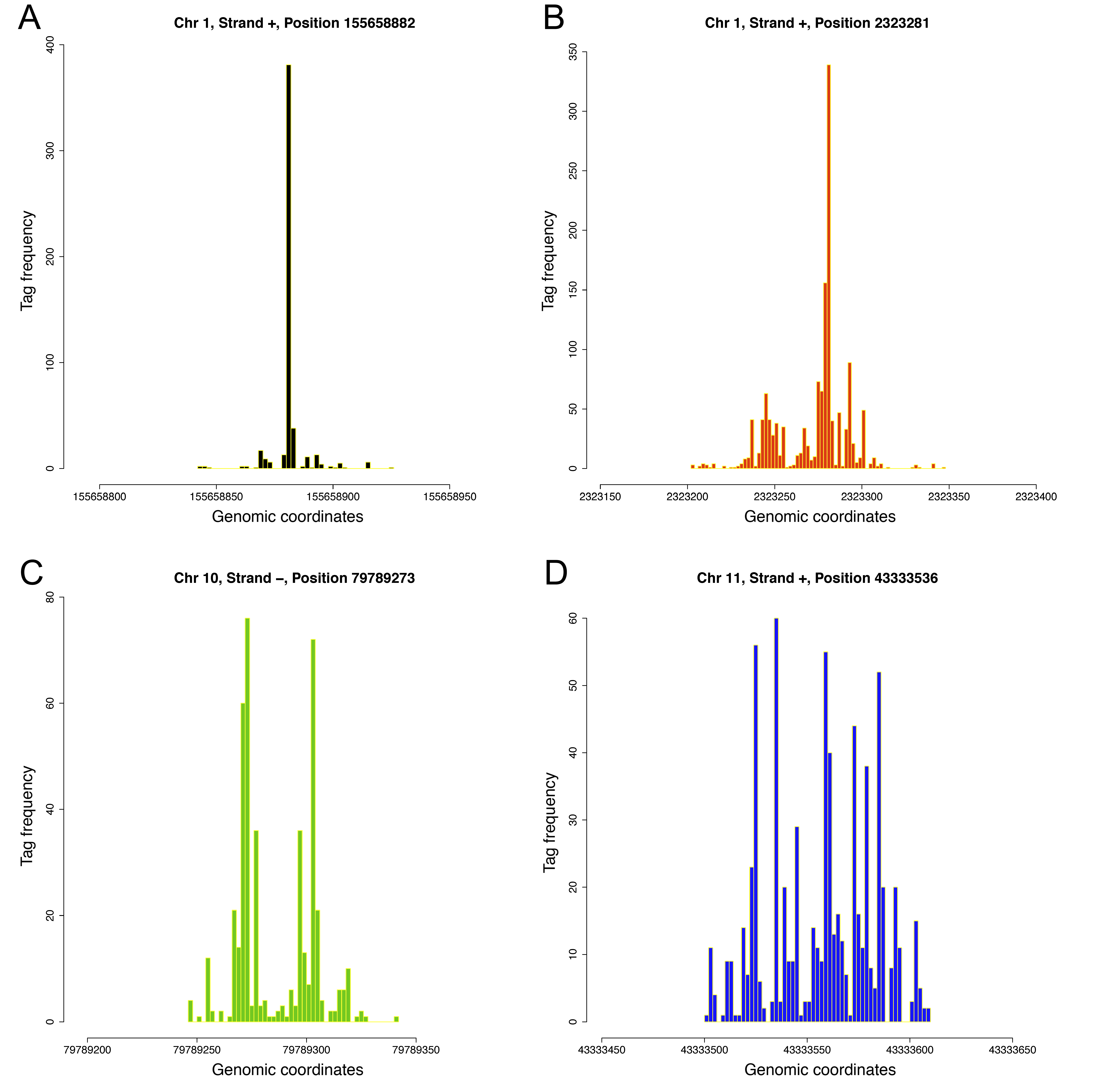

Supplement: Supplementary file 2 — Figure S2. Typical examples for the 4 TC shape classes. SP class (A) are characterized by a sharp peak that stands for the majority of tags in a TC. BP class (D) do not have any peak much stronger than the others in a TC. The 5’ end distributions in DP (B) and MP (C) classes are somewhere between SP and BP classes (refer to Methods for more details). The TC information (chromosome, strand and genomic position) are placed on top of each example. (PNG 336 kb) [file 12864_2019_5650_MOESM2_ESM.png]
