# Supplementary material for: Selective translational usage of TSS and core promoters revealed by translatome sequencing
Source: BMC Genomics. 2019 Apr 11;20:282. doi: 10.1186/s12864-019-5650-0 (PMC6463679; doi:10.1186/s12864-019-5650-0)
Supplement: Supplementary file 7 — Calculation of TC fold change (FC) with polysome-free RNA instead of total RNA. (DOCX 13 kb) [file 12864_2019_5650_MOESM7_ESM.docx]

**Supplementary Text**

**Calculation of TC fold change (FC) with polysome-free RNA instead of total RNA**

In this study, we used total RNA and polysome-associated RNA to calculate FC, which was defined as follows:

$$FC=\frac{{RPM}_{translatome}}{{RPM}_{transcriptome}}$$

If total RNA was replaced by polysome-free RNA as input material for the CAGE library preparation, we would calculate FC with the following definition:

$${FC}_{2}=\frac{{RPM}_{translatome}}{{RPM}_{polysome-free RNA}}$$

Since ${RPM}_{transcriptome}={RPM}_{translatome}+{RPM}_{polysome-associated RNA}$, we could have the following derivation:

$$FC=\frac{{RPM}_{translatome}}{{RPM}_{transcriptome}}$$

$$=\frac{{RPM}_{translatome}}{{RPM}_{translatome}+{RPM}_{polysome-free RNA}}$$

$$=\frac{{RPM}_{translatome}/{RPM}_{polysome-free RNA}}{\frac{{RPM}_{transcriptome}}{{RPM}_{polysome-free RNA}}+1}$$

$$=\frac{{FC}_{2}}{{FC}_{2}+1}$$

From the above equation, we can see that, when FC_2_ becomes larger, FC becomes larger as well. Therefore, even though there was no CAGE data from polysome-free RNA in this study, we could still know the FC_2_-based rank of TC enrichment, which should be the same as that in Table S4.
